# Effect of thyrotropin‐releasing hormone stimulation testing on the oral sugar test in horses when performed as a combined protocol

**DOI:** 10.1111/jvim.15601

**Published:** 2019-08-20

**Authors:** Elizabeth Hodge, Alycia Kowalski, Catherine Torcivia, Sue Lindborg, Darko Stefanovski, Kelsey Hart, Nicholas Frank, Andrew van Eps

**Affiliations:** ^1^ School of Veterinary Medicine University of Pennsylvania, Clinical Studies‐New Bolton Center, Kennett Square Chester Pennsylvania; ^2^ Department of Large Animal Medicine, College of Veterinary Medicine University of Georgia Athens Georgia; ^3^ Department of Clinical Sciences Tufts Cummings School of Veterinary Medicine North Grafton Massachusetts

**Keywords:** ACTH, EMS, equine Cushing's, equine metabolic syndrome, insulin dysregulation, pituitary pars intermedia dysfunction, PPID

## Abstract

**Background:**

The use of parallel dynamic tests to identify insulin dysregulation (ID) and pituitary pars intermedia dysfunction (PPID) in horses could have better diagnostic utility than measuring baseline hormone concentrations, if the tests do not alter diagnostic interpretation of one another.

**Hypothesis:**

Performing a thyrotropin‐releasing hormone (TRH) stimulation test before an oral sugar test (OST) would not affect results of OST.

**Animals:**

Twenty‐six healthy university‐owned horses.

**Methods:**

A prospective randomized placebo‐controlled, crossover design was used to evaluate 3 OST protocols: OST alone, TRH followed by OST (TRH + OST), and placebo followed by OST (placebo + OST). Agreement for plasma insulin concentrations and diagnostic interpretation were assessed with Bland‐Altman and logistic regression analyses, respectively.

**Results:**

Bland‐Altman analysis of TRH + OST versus OST alone showed good agreement between testing protocols, with bias ± SD for insulin concentrations at baseline 0.4 ± 4.7 μIU/mL (95% limits of agreement [LOA], −8.8 to 9.7), 60 minute −0.5 ± 22.6 μIU/mL (95% LOA, −44.7 to 43.8), and 90 minute 1.9 ± 20.6 μIU/mL (95% LOA, −38.5 to 42.4) after OST, similar to placebo + OST versus OST alone. Diagnostic interpretation (positive/negative) was not different between protocols (TRH + OST versus OST alone [*P* = .78], placebo + OST versus OST alone [*P* = .77], or TRH + OST versus placebo + OST [*P* = .57]).

**Conclusions and Clinical Importance:**

Concurrent testing for PPID and ID with a TRH stimulation test before an OST is an acceptable diagnostic tool for investigation of endocrinopathies in horses and allows accurate testing to be performed efficiently in 1 visit.

**Abbreviations**
EDTA: ethylenediaminetetraacetic acid
EMS: equine metabolic syndrome
ID: insulin dysregulation
OST: oral sugar test
PPID: pituitary pars intermedia dysfunction
TRH: thyrotropin‐releasing hormone

## 1 INTRODUCTION

Endocrinopathic laminitis (associated with equine metabolic syndrome [EMS] or pituitary pars intermedia dysfunction [PPID]) is the most common form of laminitis in horses.^1^, ^2^, ^3^, ^4^ However, greater than 50% of endocrinopathic laminitis cases might go unnoticed by owners.^5^ Therefore, early diagnostic testing should be employed to identify horses with underlying endocrinopathies to implement treatment and management strategies that prevent the development or progression of laminitis.

PPID is a progressive disease for which diagnostic screening tests and their reference ranges have largely been developed using horses with advanced PPID, as evidenced by multiple clinical signs that include hypertrichosis as the diagnostic criterion. Consequently, resting plasma ACTH and the associated reference ranges can yield false‐negative results in the earlier stages of the disease.^6^, ^7^, ^8^ Measurement of resting plasma ACTH concentration has low sensitivity for diagnosing PPID with substantial overlap between affected and unaffected horses especially during nonautumnal seasons.^9^, ^10^ However, the thyrotropin‐releasing hormone (TRH) stimulation test can be used for early detection of PPID in horses with resting ACTH concentrations that fall within normal reference ranges.^11^

Insulin dysregulation (ID) is a key risk factor for the development of laminitis in horses with EMS and PPID.^3^, ^7^, ^12^ Hyperinsulinemia in particular appears to be the crucial event that leads to lamellar weakening, damage, and ultimately laminitis in these cases.^13^, ^14^ The presence of hyperinsulinemia in horses with PPID is a common finding and is a prognostic indicator.^7^, ^15^ Insulin resistance is not a prerequisite for hyperinsulinemia in horses or ponies, and testing the insulin response to ingested nonstructural carbohydrate is a more physiologically relevant means of identifying hyperinsulinemia that poses a risk for laminitis development and progression.^16^ The oral sugar test (OST) can be used to evaluate the insulin response to an oral carbohydrate load, taking into account the enteroinsular axis, and has a higher sensitivity for the diagnosis of ID than does measurement of resting serum insulin concentration.^16^, ^17^, ^18^

It is often clinically indicated to test for both PPID and ID in the same animal, making a protocol that temporally combines an OST with a TRH stimulation test highly desirable for efficient clinical testing in animals with suspect PPID or ID or both, particularly in a field setting. Unfortunately, performing an OST before a TRH stimulation test blunts ACTH results after stimulation, resulting in false‐negative PPID tests, precluding the use of a combined protocol with this order of testing.^19^ The reverse order of testing has not yet been evaluated, and the aim of this study was to evaluate the effects of a TRH stimulation test on the results of an OST performed after as part of a combined testing protocol. We hypothesized that performing a TRH stimulation test before an OST would not affect OST results.

## 2 MATERIALS AND METHODS

### 2.1 Horses

Twenty‐six university‐owned horses were enrolled in a randomized placebo‐controlled crossover experimental study. All horses were apparently healthy based on physical examinations performed before each testing protocol and none of the horses had previously been tested for endocrinopathies. Seven geldings and 19 mares aged 5‐21 years (median age 10.5 years) were used and included 14 Thoroughbreds, 9 Standardbreds, 1 Morgan, 1 Friesian, and 1 Trakehner.

All horses were housed at the University of Pennsylvania New Bolton Center and offered ad libitum timothy hay and water for the entire study period. Between testing days, the horses were turned out on dry lots (n = 23) or paddocks that had minimal grass (n = 3). Three horses had their diets supplemented with a pelleted ration (Triple Crown Senior, Triple Crown Nutrition, Inc., Wayzata MN) because of a historical difficulty maintaining body condition on a solely hay diet.

### 2.2 Experimental design

All experimental procedures were approved by the University of Pennsylvania's Institutional Animal Care and Use Committee. Three testing protocols were carried out with a 3‐day wash‐out period in between each test for each horse: an OST (OST alone), TRH stimulation test followed by an OST (TRH + OST), and a saline injection followed by OST (placebo + OST). A baseline plasma ACTH concentration was measured along with the baseline insulin and glucose concentrations as part of each testing protocol. The order of testing protocols was incompletely blocked with horses randomly assigned to the testing protocols to account for the effect of multiple OST (Table S1). An a priori sample size power calculation to estimate the number of animals needed to detect a significant difference in OST results among test protocols was guided by results of a prior study that enabled estimation of the expected variance of the mean difference in insulin concentration between test protocols (an SD of 10 μIU/mL for OST insulin was used in calculations based on the previous data).^20^ Twenty‐four horses were required to provide 80% power to detect a difference in insulin concentration of 15 μIU/mL, with a significance level (alpha) of .05. Based on these results, 26 horses were enrolled in the event that 1‐2 horses could not complete the study.

During the period of February 28th to March 10th, 2018, horses were tested in groups of 8‐9 at a time, with all testing carried out between 5:30 am‐9 am The horses were brought into stalls the afternoon before testing and had ad libitum timothy hay and water until 8 hours before testing, at which time muzzles were placed to restrict feed consumption while still allowing access to water. Before each test protocol physical examination, weight, body condition score (BCS), and cresty neck score (CNS) were recorded for each horse.^21^, ^22^ These assessments were performed each time the horses were brought into stalls by 2 veterinarians (EH and AK). For logistical reasons related to stabling locations, 14 horses were weighed on a scale and 12 horses were weighed with the use of a weight tape (Horse & Pony Height Weight Tape, The Coburn Company, Inc., Whitewater WI).

### 2.3 Endocrine test protocols

OST was performed in all 3 testing protocols as previously described by Schuver et al.^17^ Briefly, corn syrup (Karo Light Corn Syrup, ACH Food Companies, Inc., Oakbrook Terrace IL) was administered at 0.15 mL/kg body weight with an oral dosing syringe. Blood was collected into ethylenediaminetetraacetic acid (EDTA) and sodium fluoride blood tubes (BD vacutainer blood collection tubes, Becton, Dickinson and Company, Franklin Lakes, NJ) before and at 60 and 90 minutes after administration of corn syrup.

Synthetic TRH (≥98% [HPLC], powder, Sigma‐Aldrich Co, St Louis MO) was reconstituted with sterile 0.9% saline to a 1 mg/mL solution using sterile technique under a biosafety hood and filtered through a 0.22 μm syringe filter. TRH was distributed into 1 mL aliquots and stored at −80°C until the time of use. The combined TRH stimulation and OST was performed by drawing a blood sample into EDTA and sodium fluoride blood tubes for baseline plasma ACTH, insulin, and glucose concentrations (same as for the OST alone) before administering 1 mg of TRH IV via direct jugular venipuncture. A second 7 mL blood sample was collected into an EDTA tube at 10 minutes after TRH administration for plasma ACTH concentration. After the second blood draw, corn syrup was administered for the OST as described above, and subsequent blood samples for plasma insulin and glucose concentrations were drawn 60 and 90 minutes after corn syrup administration (approximately 70 and 100 minutes after the baseline sample). The combined placebo and OST protocol was performed in the same manner, with 1 mL 0.9% saline administered IV in the place of TRH. Horses were returned to their paddocks after the 90‐minute blood sample.

Each OST was classified as positive or negative (OST outcome) based on current accepted cutoffs for ID in horses.^19^, ^20^ A fasting baseline insulin concentration ≥ 20 μIU/mL or insulin concentration at 60 or 90 minutes after oral sugar ≥45 μIU/mL was considered a positive outcome for the OST when measurement of insulin alone was used. When both insulin and glucose were used, baseline insulin concentration ≥ 20 μIU/mL, after OST insulin concentration ≥ 45 μIU/mL, or a glucose >125 mg/dL at any time was considered a positive result for the OST. The reference ranges provided by the laboratory for resting plasma ACTH and 10 minutes after TRH ACTH of >35 pg/mL and > 110 pg/mL, respectively, were used for the classification of PPID as positive or negative.

### 2.4 Plasma ACTH, insulin, and glucose

All samples were centrifuged within 4 hours, plasma separated into 2 mL plastic tubes, and stored at −80°C until analysis. EDTA plasma samples were submitted to Cornell University's Endocrinology Laboratory (Cornell University Animal Health Diagnostic Center, Ithaca NY) for ACTH and insulin concentrations measured by validated chemiluminescent immunoassay (Immulite ACTH chemiluminescent assay, Siemens Medical Solutions Diagnostics, Los Angeles, CA) and radioimmunoassay (Millipore Sigma, Merck KGaA, Darmstadt, Germany), respectively.^6^, ^23^ Sodium fluoride plasma was submitted to the Cornell University Animal Health Diagnostic Center (Ithaca, NY) for the measurement of plasma glucose concentrations via hexokinase method (Roche Cobas c501 analyzer, Roche Diagnostics, Indianapolis, IN). All samples were sent on ice overnight and were reported to be frozen upon arrival.

### 2.5 Statistical analysis

Data were assessed visually and by performing Shapiro‐Wilk tests for normality. Median and interquartile range (IQR) values were reported. Multilevel mixed‐effects linear regression (with random effects set at the level of the individual horse and robust estimation of variance) was used to evaluate for effect of test protocol, test order, ACTH, and age on insulin and glucose concentrations. Pairwise Bland‐Altman tests were performed to evaluate agreement between the different test protocols for insulin concentrations. Logistic regression adjusted for confounders evaluated for binary (positive/negative) outcome between test protocols. The agreement (%) between the test protocols for positive/negative OST results was also calculated. All analyses were conducted using commercial software (GraphPad Prism 7, GraphPad software, La Jolla CA and Stata 15.1MP, StataCorp, State College TX) with 2‐sided tests of hypotheses and a *P*‐value <.05 as the criterion for statistical significance.

## 3 RESULTS

### 3.1 Horses

No complications developed in any of the 26 horses during the study period. Body weight ranged from 491‐671 kg with a median of 538 kg. The mean and SD of BCS and CNS were 5.9 ± 1.0 and 1.4 ± 0.8, respectively. Data for individual horses including age, sex, breed, weight, BCS, CNS, PPID classification, ID classification, and mean plasma ACTH, insulin, and glucose are reported in Tables S2 and S3.

### 3.2 Plasma ACTH, insulin, and glucose

**Figure 1:**
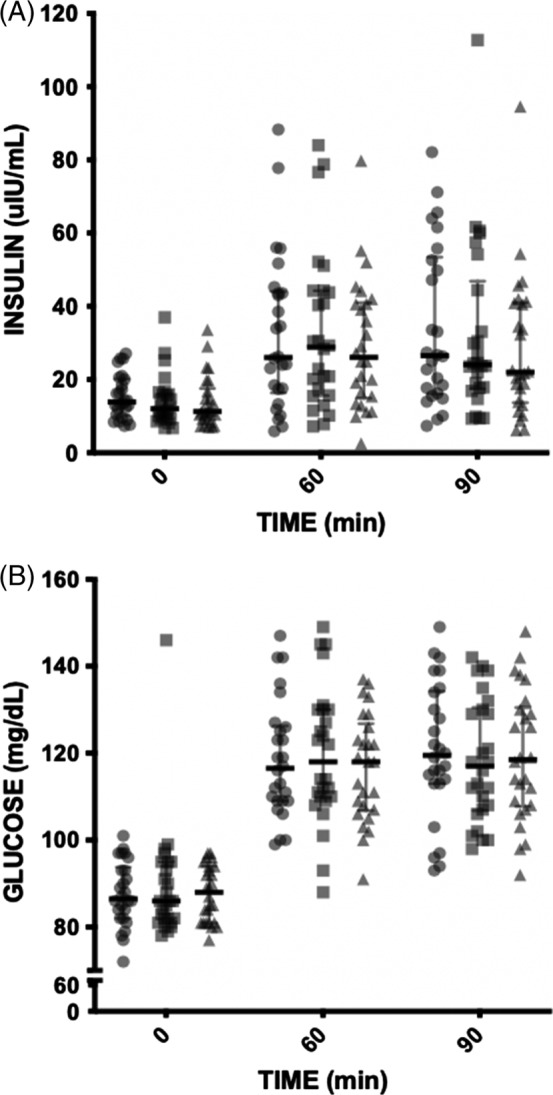
Insulin (A) and glucose (B) concentrations at baseline, 60 minute, and 90 minute for the 3 test protocols with median and interquartile range shown. Circles represent values from the OST alone protocol, squares represent values from the TRH + OST protocol, and triangles represent values from the placebo + OST protocol

Median (IQR) plasma ACTH, insulin, and glucose concentrations from the 3 protocols as well as test number are shown in Table 1 and Table S4. Two horses had baseline ACTH >35 pg/mL on 2 occasions but ACTH 10 minutes after TRH was ≤110 pg/mL in both horses. Two horses had an ACTH >110 pg/mL 10 minutes after TRH administration. Of those 2 horses, 1 had a resting ACTH >35 pg/mL on only 1 occasion. Plasma insulin concentration was significantly affected by both baseline ACTH concentration and age: higher baseline ACTH concentration (regression model coefficient [Coeff.] .18, 95% CI .08‐.28; *P* = .001) as well as older age (Coeff. 1.24, 95% CI .49‐1.99; *P* = .001) were associated with higher insulin concentrations. Plasma glucose was also significantly affected by baseline ACTH (Coeff. 0.34, 95% CI .18‐.49; *P* < .001). Median (IQR) plasma insulin and glucose concentrations from each of the 3 protocols are plotted in Figure 1. Testing protocol did not have a significant effect on insulin or glucose concentrations between the OST alone protocol compared to the TRH + OST protocol for insulin (*P* = .64) or glucose (*P* = .92), the OST alone protocol compared to the placebo + OST protocol for insulin (*P* = .21) or glucose (*P* = .66), or the placebo + OST protocol compared to the TRH + OST for insulin (*P* = .20) or glucose (*P* = .73). Because horses were tested with 3 OST protocols in different orders, the effect of test order regardless of protocol was evaluated. Test order had a significant effect when results from the first test were compared to the third test for insulin (*P* = .02) but not glucose concentrations (*P* = .33). No significant effect of test order was observed between the first test and the second test for insulin (*P* = .40) or glucose (*P* = .35) or between the second test and the third test for insulin (*P* = .35) or glucose (*P* = .94).

**Table 1:** Median (interquartile range) baseline ACTH, insulin and glucose concentrations, insulin and glucose concentrations 60 minutes after oral sugar administration, and insulin and glucose concentrations 90 minutes after oral sugar administration from each of the 3 oral sugar test protocols

	OST alone	TRH + OST	Placebo + OST
ACTH (pg/mL)			
Baseline	18.1 (15.6‐21.2)	17.0 (14.7‐20.9)	17.1 (13.0‐22.2)
10 min after TRH/placebo	—	36.2 (26.7‐46.4)	18.5 (15.5‐24.1)
Insulin (uIU/mL)			
Baseline	13.9 (10.3‐20.2)	13.6 (10.0‐17.3)	12.7 (9.3‐17.6)
60 min after OST	26.0 (16.4‐44.0)	28.8 (15.6‐44.3)	26.1 (14.9‐41.0)
90 min after OST	26.6 (16.1‐53.4)	24.1 (17.5‐46.9)	22.0 (13.7‐41.0)
Glucose (mg/dL)			
Baseline	86.5 (82.0‐93.8)	86.0 (81.8‐95.0)	88.0 (81.0‐94.0)
60 min after OST	116.5 (109.0‐126.3)	118.0 (109.8‐130.0)	118.0 (106.8‐126.8)
90 min after OST	119.5 (113.0‐134.3)	117.0 (106.8‐130.5)	118.5 (107.8‐130.5)

### 3.3 Bland‐Altman analysis

**Figure 2:**
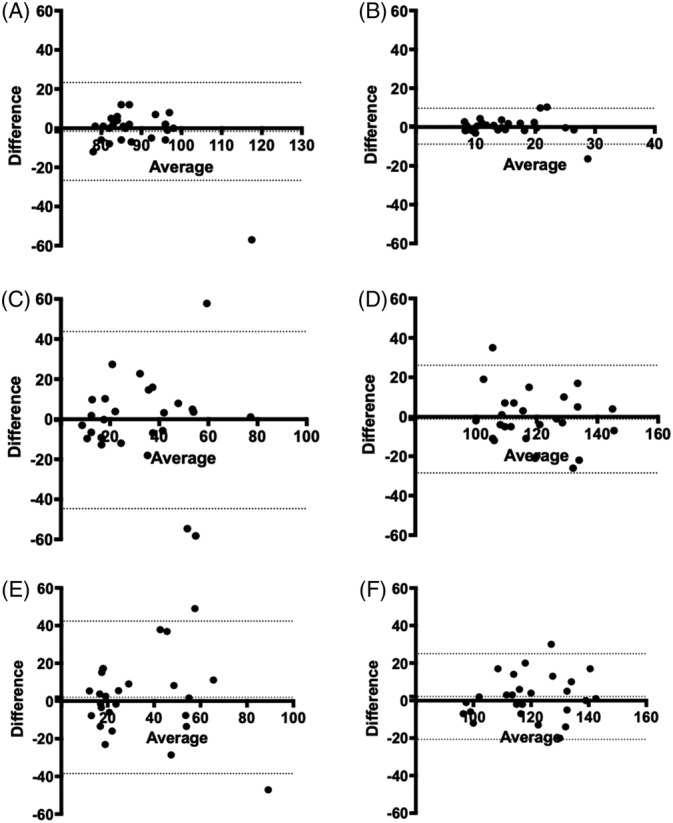
Bland‐Altman difference versus average plots comparing results from TRH + OST and OST alone for baseline plasma insulin (A) and glucose (B) concentrations, plasma insulin (C) and glucose (D) concentrations 60 minutes after oral sugar administration, and plasma insulin (E) and glucose (F) concentrations 90 minutes after oral sugar administration

Difference versus average plots demonstrated good agreement for insulin and glucose concentrations in horses between the TRH + OST protocol and the OST alone protocols (Figure 2). Mean difference between insulin concentrations from TRH + OST and OST alone at baseline (bias .414 μIU/mL), 60 minutes (bias −.452 μIU/mL), and 90 minutes (bias 1.94 μIU/mL) were close to zero for all comparisons. Additionally, the points demonstrated random dispersion consistent with an absence of heteroskedasticity. Finally, the 95% limits of agreement (LOA) for insulin concentrations from the placebo + OST compared to OST alone were similar to the 95% LOA for TRH + OST compared to OST alone, showing no clinically significant difference in insulin concentration because of TRH administration before OST. Bland‐Altman plots for TRH + OST and placebo + OST as well as placebo + OST and OST alone are not shown. Results of Bland‐Altman analysis for insulin and glucose concentrations from all test protocol comparisons are presented in Table 2 and Table 3, respectively.

**Table 2:** Results of Bland‐Altman analysis showing bias, SD, and 95% limits of agreement (LOA) for plasma insulin concentrations at all time points

	Baseline insulin		60 min after OST insulin		90 min after OST insulin	
	Bias (SD)	95% LOA	Bias (SD)	95% LOA	Bias (SD)	95% LOA
TRH + OST versus OST alone	0.4 (4.7)	−8.8 to 9.7	−0.5 (22.6)	−44.7 to 43.8	1.9 (20.6)	−38.5 to 42.4
TRH + OST versus placebo	0.1 (4.2)	−8.1 to 8.3	3.5 (17.8)	−31.3 to 38.4	3.8 (20.3)	−36.0 to 43.5
Placebo versus OST alone	0.5 (4.1)	−7.5 to 8.5	3.1 (21.7)	−39.4 to 45.5	5.7 (23.8)	−40.9 to 52.3

**Table 3:** Results of Bland‐Altman analysis showing bias, SD, and 95% limits of agreement (LOA) for plasma glucose concentrations at all time points

	Baseline glucose		60 min after OST glucose		90 min after OST glucose	
	Bias (SD)	95% LOA	Bias (SD)	95% LOA	Bias (SD)	95% LOA
TRH + OST versus OST alone	−1.7 (12.8)	−26.7 to 23.3	−1.2 (13.9)	−28.5 to 26.1	2.2 (11.7)	−20.7 to 25.0
TRH + OST versus placebo	1.6 (11.8)	−21.6 to 24.7	3.0 (10.5)	−17.6 to 23.6	−1.1 (9.3)	−19.3 to 17.1
Placebo versus OST alone	−0.1 (5.7)	−11.3 to 11.2	1.8 (11.3)	−20.4 to 24.0	1.0 (12.9)	−24.2 to 26.3

### 3.4 Oral sugar test diagnostic interpretation

**Figure 3:**
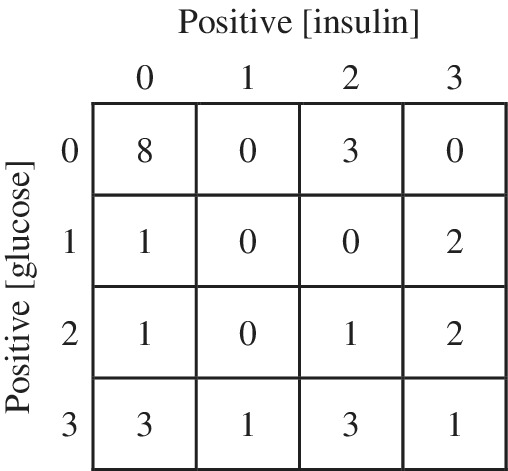
The number of horses classified as positive based on glucose or insulin on 3, 2, 1, or none of the 3 oral sugar tests is represented inside each box

Of the 26 horses, 18 had OST outcomes classified as positive on at least 1 occasion when both insulin and glucose concentrations were evaluated from the 3 OSTs performed. When insulin alone was used to classify OST outcome, 13 horses were considered positive. The number of horses classified as positive or negative by insulin or glucose concentrations by the 3 OSTs is summarized in Figure 3. There was no statistically significant difference between testing protocols when both insulin and glucose concentrations were used to classify OST outcome as positive or negative between the TRH + OST and the OST alone protocol (*P* = .78), placebo + OST and the OST alone protocol (*P* = .77), or the TRH + OST and placebo + OST protocol (*P* = .57). Similarly, there was no significant difference in outcome when insulin alone was used to classify horses as positive or negative between test protocols.

Agreement for a positive or negative OST outcome was 81% on all 3 testing protocols when both insulin and glucose concentrations were used, 69% when only insulin concentrations were evaluated, and 73% when only glucose concentrations were evaluated. For OST alone and TRH + OST test outcome, there was a positive percent agreement of 88% and a negative percent agreement of 90%.

## 4 DISCUSSION

Performing the TRH stimulation test before the OST did not alter the insulin or glucose concentrations measured during the OST, nor did this combined testing protocol significantly alter the interpretation of the OST outcome based on current diagnostic cutoff criteria. The results indicate that this combined protocol is suitable for clinical diagnostic use in a population of horses with equivocal or no clinical signs of PPID, similar to the current study. This combined testing protocol requires approximately 105 minutes to complete and utilizes dynamic testing to provide relevant information for the diagnosis of both PPID and ID. Early and accurate diagnosis is important as horses with PPID or ID can develop laminitis as the presenting first sign.^4^, ^5^, ^11^, ^18^

Several studies have previously evaluated the repeatability of the OST.^17^, ^20^, ^24^ Initial studies showed poor repeatability for insulin and glucose concentrations after the relatively low dose of sugar used in the OST.^17^, ^24^ A more recent study found acceptable repeatability and good agreement for insulin but not glucose when comparing positive/negative interpretations of the OST performed on 2 different occasions in 53 horses.^20^ In the current study, Bland‐Altman analysis of insulin concentrations showed that the agreement between the TRH + OST and OST alone protocols is similar to what would be expected for the agreement between 2 OSTs performed alone on different days, demonstrating that this combined testing protocol did not cause clinically relevant differences in the measured variables. Additionally, the current study Bland‐Altman analysis for plasma glucose concentration demonstrated good agreement at baseline as well as 60 and 90 minutes after oral sugar administration between each of the 3 test protocols with mean bias close to zero, lower SD, and a smaller range for 95% LOA than previously reported for OST performed on 2 different days.^20^ This is important as the cutoffs for diagnosing ID are continuing to evolve.^18^, ^25^, ^26^

Assessment of the agreement for diagnostic interpretation of OST results was performed because of the previously reported high variability in absolute values of plasma insulin and glucose concentrations on different days.^24^, ^27^ However, the current study was not designed to evaluate the diagnostic accuracy of the OST nor the TRH stimulation test. Values used to assign a positive diagnosis were based on current clinical recommendations, were consistent with previous studies, and were set as the reference ranges provided by the laboratory.^18^, ^19^, ^20^, ^28^, ^29^ Binary (positive/negative) test outcomes from the TRH + OST and OST alone test protocols evaluating insulin concentrations alone, as well as both insulin and glucose concentrations, demonstrated equivalence between the test protocols.

While none of the horses used in this study had previously been tested for or diagnosed with PPID, 4 horses had plasma ACTH concentrations that exceeded the upper limit of current laboratory reference range for PPID. Two horses (ages 13 and 15 years old) with normal ACTH after TRH stimulation test had increased resting ACTH concentrations on 2 of the 3 testing occasions. It is possible that these horses had increased resting ACTH concentrations because of stress, or false‐negative after TRH ACTH levels.^30^, ^31^, ^32^ Currently accepted reference ranges for ACTH reflect a seasonal increase in the fall but not a seasonal decrease in the spring despite previous studies demonstrating lower resting as well as after TRH ACTH concentrations in the spring.^31^, ^33^, ^34^, ^35^ Regardless of the reason, the low number of horses with increased ACTH, especially after the TRH stimulation test, precludes any conclusion from being drawn concerning the effect of high ACTH after TRH on OST outcome. Horses with PPID and ID have higher endogenous ACTH concentrations than do horses with PPID and no evidence of ID.^36^ A similar relationship was appreciated with increasing insulin concentrations observed as resting ACTH concentrations increased in the current study, although small numbers preclude accurate conclusions from being drawn on the effect of ACTH concentration on OST results. Further studies are needed to evaluate the effect of combined testing in this manner on horses with PPID and markedly increased circulating ACTH levels. Additionally, more information is needed to evaluate for a seasonal influence on combined endocrine testing in this manner.

Another limitation of this study is that the OST protocols were performed every 4 days and the effect of this short interval on repeat testing is not established. This testing interval was utilized to ensure that all 3 tests were performed within 2 weeks to minimize any possible effects of season or diet changes for horses housed on pasture with grass. Currently, there is no recommended “wash‐out” period between OSTs, and the effect of frequent OSTs on subsequent OST results is not known. Diets high in nonstructural carbohydrates have been shown to increase the insulin response to sugar compared to diets high in fat.^37^ However, the isolated doses of corn syrup administered in this study were considered unlikely to have had a similar type of effect. Despite this, insulin concentrations from the third test were significantly higher than the first test. While the study design accounted for possible effects of multiple oral sugar tests by randomly assigning horses to receive the test protocols in different orders, future studies could consider increasing the amount of time between OSTs.

The OST + placebo protocol was included in this study because of the concern that the additional stress associated with performing the TRH test (administration and sampling, rather than the TRH itself) might affect OST results.^38^, ^39^ No significant difference in OST results was appreciated when the placebo + OST protocol was compared to the OST alone, demonstrating that the stress associated with the additional venipunctures did not affect 60 or 90 minute glucose or insulin concentrations. Stress of hospitalization/procedures could affect insulin dynamics by increasing the release of stress hormones.^29^, ^38^, ^39^, ^40^ Glucocorticoids and catecholamines decrease insulin sensitivity in horses.^41^, ^42^ One possible explanation for the increased insulin concentrations appreciated during the third OST could be because of the effect of stress from multiple periods of fasting, environment change, and administration of each test. Stress from fasting and transport increased insulin concentrations in equids, although in another experimental model fasting did not affect cortisol levels.^43^, ^44^, ^45^ More research on the effect of environmental and handling stress on insulin concentration in horses is needed.

A primary goal for veterinarians in diagnosing and treating equine endocrine disease should be to prevent the development of laminitis through early detection of endocrine disorders that place horses and ponies at‐risk for laminitis. Dynamic testing with TRH stimulation test and OST is currently the most widely accepted method available for early diagnosis of these conditions in horses, but a protocol for combining these tests into 1 visit without negatively impacting the results was not previously available. Owners and veterinarians are more likely to test for both PPID and EMS if testing can be combined efficiently in 1 visit. The results of this study support the use of combined testing for PPID and ID by performing the TRH stimulation test before the OST, although further validation of these findings in a cohort of horses with confirmed PPID and ID is recommended.

## CONFLICT OF INTEREST DECLARATION

Authors declare no conflict of interest.

## OFF‐LABEL ANTIMICROBIAL DECLARATION

Authors declare no off‐label use of antimicrobials

## INSTITUTIONAL ANIMAL CARE AND USE COMMITTEE (IACUC) OR OTHER APPROVAL DECLARATION

Approval obtained from the University of Pennsylvania's IACUC (protocol # 806446).

## HUMAN ETHICS APPROVAL DECLARATION

Authors declare human ethics approval was not needed for this study.

## Supporting information


**Supplementary 1** Testing schedule using an incompletely blocked randomized placebo‐controlled crossover experimental design.

**Supplementary 2** Data for the individual horses age, sex, weight, BCS, CNS, and PPID/ ID classification is presented. Classification for PPID based on baseline ACTH >35 pg/mL or after TRH ACTH >110 pg/mL, and classification for ID based on baseline insulin concentration ≥ 20 μIU/mL, after OST insulin concentration ≥ 45 μIU/mL, or a glucose >125 mg/dL at any time.

**Supplementary 3** Mean and range for ACTH, insulin, and glucose concentrations from the 3 testing occasions reported for individual horses.

**Supplementary 4** Median (interquartile range) baseline ACTH, insulin and glucose concentrations, insulin and glucose concentrations 60 minutes after oral sugar administration, and insulin and glucose concentrations 90 minutes after oral sugar administration from each of the 3 OST.
