# Supplementary material for: Effect of thyrotropin‐releasing hormone stimulation testing on the oral sugar test in horses when performed as a combined protocol
Source: J Vet Intern Med. 2019 Aug 20;33(5):2272–9. doi: 10.1111/jvim.15601 (PMC6766522; doi:10.1111/jvim.15601)
Supplement: Supplementary file 1 — Supplementary 1 Testing schedule using an incompletely blocked randomized placebo‐controlled crossover experimental design. [file JVIM-33-2272-s001.pdf]

HORSE	TEST 1	Date of 1st TEST	TEST 2	Date of 2nd TEST	TEST 3	Date of 3rd TEST
1	OST	28-Feb	TRH+OST	4-Mar	Saline+OST	8-Mar
2	TRH+OST	28-Feb	Saline+OST	4-Mar	OST	8-Mar
3	Saline+OST	28-Feb	OST	4-Mar	TRH+OST	8-Mar
4	TRH+OST	28-Feb	OST	4-Mar	Saline+OST	8-Mar
5	OST	28-Feb	TRH+OST	4-Mar	Saline+OST	8-Mar
6	TRH+OST	28-Feb	Saline+OST	4-Mar	OST	8-Mar
7	Saline+OST	28-Feb	OST	4-Mar	TRH+OST	8-Mar
8	TRH+OST	28-Feb	OST	4-Mar	Saline+OST	8-Mar
9	OST	28-Feb	Saline+OST	4-Mar	TRH+OST	8-Mar
10	OST	1-Mar	Saline+OST	5-Mar	TRH+OST	9-Mar
11	TRH+OST	1-Mar	OST	5-Mar	Saline+OST	9-Mar
12	Saline+OST	1-Mar	TRH+OST	5-Mar	OST	9-Mar
13	OST	1-Mar	Saline+OST	5-Mar	TRH+OST	9-Mar
14	TRH+OST	1-Mar	OST	5-Mar	Saline+OST	9-Mar
15	Saline+OST	1-Mar	TRH+OST	5-Mar	OST	9-Mar
16	OST	1-Mar	Saline+OST	5-Mar	TRH+OST	9-Mar
17	OST	1-Mar	TRH+OST	5-Mar	Saline+OST	9-Mar
18	TRH+OST	1-Mar	Saline+OST	5-Mar	OST	9-Mar
19	OST	2-Mar	TRH+OST	6-Mar	Saline+OST	10-Mar
20	TRH+OST	2-Mar	Saline+OST	6-Mar	OST	10-Mar
21	Saline+OST	2-Mar	OST	6-Mar	TRH+OST	10-Mar
22	Saline+OST	2-Mar	TRH+OST	6-Mar	OST	10-Mar
23	Saline+OST	2-Mar	OST	6-Mar	TRH+OST	10-Mar
24	Saline+OST	2-Mar	TRH+OST	6-Mar	OST	10-Mar
25	OST	2-Mar	Saline+OST	6-Mar	TRH+OST	10-Mar
26	TRH+OST	2-Mar	OST	6-Mar	Saline+OST	10-Mar

Supplementary 1: Testing schedule using an incompletely blocked randomized placebo-controlled crossover experimental design.
