# Supplementary material for: Effect of thyrotropin‐releasing hormone stimulation testing on the oral sugar test in horses when performed as a combined protocol
Source: J Vet Intern Med. 2019 Aug 20;33(5):2272–9. doi: 10.1111/jvim.15601 (PMC6766522; doi:10.1111/jvim.15601)
Supplement: Supplementary file 2 — Supplementary 2 Data for the individual horses age, sex, weight, BCS, CNS, and PPID/ ID classification is presented. Classification for PPID based on baseline ACTH >35 pg/mL or after TRH ACTH >110 pg/mL, and classification for ID based on baseline insulin concentration ≥ 20 μIU/mL, after OST insulin concentration ≥ 45 μIU/mL, or a glucose >125 mg/dL at any time. [file JVIM-33-2272-s002.pdf]

Horse	Age	Sex	Breed	Weight (kg)	Mean BCS	Mean CNS	PPID classification	# binary positive classification OST- insulin only	# binary positive classification OST - glucose only	# binary positive classification OST
1	20	F	TB	539	6.2	1.6	+	2	3	3
2	17	F	TB	590	6.8	2.6	-	2	3	3
3	21	F	TRAK	553	5.8	1.4	+	3	2	3
4	15	F	TBx	526	5.6	1	+	1	3	3
5	8	F	SB	525	5.3	1.2	-	0	1	1
6	9	G	TB	634	5.7	1.3	-	0	0	0
7	7	F	TB	546	4	0.3	-	0	3	3
8	13	F	TB	504	5.3	0.8	-	0	3	3
9	8	G	SB	671	6.5	2.8	-	2	0	2
10	18	F	SB	493	6.3	1.5	-	2	0	2
11	13	F	TB	514	4.6	0.3	-	0	0	0
12	8	F	SB	491	5.6	1.3	-	0	0	0
13	13	F	TBx	593	6.7	2.3	-	3	1	3
14	6	G	TB	536	4.6	0.5	-	0	0	0
15	10	F	TB	597	6.3	0.8	-	2	3	3
16	9	G	SB	516	5.2	0.9	-	0	0	0
17	8	G	SB	560	6.8	2.2	-	0	3	3
18	13	F	TB	529	5	1	-	2	0	2
19	10	F	SB	605	6.8	1.2	-	3	2	3
20	19	F	Morgan	535	7.5	2.1	-	3	3	3
21	17	F	TB	571	6.9	2.8	-	3	1	3
22	13	F	SB	539	6.9	1.8	+	0	0	0
23	5	F	Fresian	598	8	3.2	-	2	2	3
24	5	G	TB	532	4.3	0.2	-	0	0	0
25	6	F	SB	516	6.2	1.5	-	0	2	2
26	11	G	TB	510	4.7	1	-	0	0	0

Supplementary 2: Data for the individual horses age, sex, weight, BCS, CNS, and PPID/ ID classification is presented. Classification for PPID based on baseline ACTH > 35 pg/mL or after

TRH ACTH > 110 pg/mL, and classification for ID based on baseline insulin concentration ≥ 20 μIU/mL, after OST insulin concentration ≥ 45 μIU/mL, or a glucose > 125 mg/dL at any time.
