# Supplementary material for: Effect of thyrotropin‐releasing hormone stimulation testing on the oral sugar test in horses when performed as a combined protocol
Source: J Vet Intern Med. 2019 Aug 20;33(5):2272–9. doi: 10.1111/jvim.15601 (PMC6766522; doi:10.1111/jvim.15601)
Supplement: Supplementary file 3 — Supplementary 3 Mean and range for ACTH, insulin, and glucose concentrations from the 3 testing occasions reported for individual horses. [file JVIM-33-2272-s003.pdf]

Horse	Baseline ACTH mean (range)	Baseline insulin mean (range)	Baseline glucose mean (range)	60 min insulin mean (range)	60 min glucose mean (range)	90 min insulin mean (range)	90 min glucose mean (range)
1	17.4 (15.6-18.6)	14.68 (13-16.54)	85 (80-89)	33.16 (24.91-45.27)	127 (122-134)	29.17 (14.7-54.35)	138 (130-148)
2	18 (13.2-22.9)	17.39 (15.79-19.1)	93 (90-95)	45.36 (40.58-51.71)	136 (119-147)	49.26 (40.59-57.43)	139 (139-139)
3	56.4 (29.7-105)	22.15 (18.63-26.8)	110 (94-146)	51.7 (25.77-83.99)	127 (118-145)	45.56 (33.05-61.66)	129 (120-140)
4	36.4 (29.6-41.7)	13.63 (12.72-14.81)	96 (92-98)	21.28(9.1-39.1)	135 (123-145)	26.75 (15.54-45.71)	129 (118-139)
5	16.1 (14.2-18.8)	13.54 (11.92-16.18)	84 (82-86)	38.92 (33.96-41.99)	120 (111-126)	20.93 (13.95-29.88)	114 (107-121)
6	14.2 (11.5-16.2)	9.46 (8.32-11.58)	81 (80-82)	10.87 (5.88-15.49)	97 (91-101)	13.35 (6.16-17.68)	101 (98-103)
7	18.1 (16.6-20.9)	11.02 (8.57-12.97)	90 (86-96)	21.72 (20.15-24.03)	129 (126-133)	19.41 (9.35-26.35)	134 (130-137)
8	14.4 (10.3-16.9)	12.69 (9.3-14.51)	94 (90-97)	20.64 (11.85-29.1)	119 (110-125)	23.55 (9.44-34.46)	140 (132-149)
9	14.1 (11.5-18.7)	19.51 (14.62-27.09)	85 (85-86)	32.42 (13.35-43.56)	107 (103-109)	41.03 (13.09- 55.79)	112 (103-119)
10	7.6 (2.9-11.6)	14.5 (13.53-15.65)	80 (77-83)	46 (38.55-55.17)	108 (106-112)	36.98 (13.91-52.64)	96 (92-98)
11	6.67 (5.8-7.5)	8.16 (7.4-8.9)	84 (81-86)	13.42 (11.51-15.43)	102 (93-112)	12.14 (9.47-17.66)	108 (100-117)
12	22.1 (14.8-27.2)	8.77 (7.52-10.29)	82 (78-86)	18.67 (12.91-23.11)	109 (106-113)	16.19 (10.28-20.39)	104 (94-112)
13	25.9 (23.8-28.5)	27.34 (25.74-29.07)	89 (79-97)	65.02 (40.67-77.75)	116 (109-123)	90.99 (65.63-112.75)	118 (110-130)
14	18.8 (17-22)	7.94 (7.24-9.22)	84 (81-87)	12.51 (2.54-17.6)	111 (109-114)	13.32 (6.32-23.55)	116 (114-119)
15	18 (16.9-18.7)	17.26 (16.52-18.45)	95 (93-99)	50.71 (44.2-55.78)	142 (134-149)	47.2 (33.78-60.66)	139 (132-143)
16	13.7 (12.1-14.6)	10.95 (9.97-12.11)	85 (81-93)	22.25 (7.23-34.59)	120 118-123)	22.7 (18.78-27.38)	118 (114-122)
17	18.9 (18.7-19)	9.91 (9.19-10.49)	79 (77-81)	32.22 (20.85-43.58)	126 (122-130)	22.31 (8.8-33.6)	121 (116-128)
18	12.1 (11.1-12.6)	15.26 (14.6-16.38)	87 (84-91)	34.56 (28.38-43.02)	106 (100-111)	44.01 (23.71-61.55)	115 (113-120)
19	18.6 (16.7-20.3)	21.22 (20.04-23.17)	97 (93-101)	32.6 (26.25-44.31)	125 (110-136)	22.3 (9.09-40.91)	129 (116-142)
20	16.5 (13.9-18.6)	27.89 (24.85-33.57)	94 (90-97)	60.89(24.16-79.74)	132 (129-136)	44.14 (27.11-64.01)	124 (112-142)
21	18.7 (16.7-20.5)	26.98 (20.58-36.99)	79 (78-80)	56.93 (30.49-88.29)	104 (88-123)	54.5 (32.36-71.14)	114 (107-128)
22	40.1 (33.3-46.1)	12.5 (11.36-13.49)	84 (81-88)	20.59 (12.89-30.43)	109 (106-111)	22.97 (21.73-24.43)	97 (93-100)
23	18.3 (16.9-21.1)	20.5 (15.94-25.79)	95 (93-97)	39.42 (11.11-56.04)	132 (125-142)	45.8 (22.23-82.11)	128 (121-134)
24	19.0 (11.7-30.2)	7.79 (6.8-9.51)	82 (72-89)	14.3 (9.86-22.89)	115 (113-117)	15.4 (9.87-25.05)	111 (106-115)
25	21 (18.6-23.7)	11.57 (11.15-12.3)	95 (92-97)	11.29 (7.14-16.54)	117 (109-130)	19.71 (7.37-30.46)	130 (125-139)
26	16.8 (15.9-18.1)	9.11 (7.61-10.47)	83 (82-84)	14.85 (7.81-19.15)	106 (100-112)	20.65 (17.7-23.75)	102 (96-109)

Supplementary 3: Mean and range for ACTH, insulin, and glucose concentrations from the 3 testing occasions reported for individual horses.
