# Supplementary material for: Effect of thyrotropin‐releasing hormone stimulation testing on the oral sugar test in horses when performed as a combined protocol
Source: J Vet Intern Med. 2019 Aug 20;33(5):2272–9. doi: 10.1111/jvim.15601 (PMC6766522; doi:10.1111/jvim.15601)
Supplement: Supplementary file 4 — Supplementary 4 Median (interquartile range) baseline ACTH, insulin and glucose concentrations, insulin and glucose concentrations 60 minutes after oral sugar administration, and insulin and glucose concentrations 90 minutes after oral sugar administration from each of the 3 OST. [file JVIM-33-2272-s004.pdf]

		1st test	2nd test	3rd test
**ACTH (pg/mL)**	Baseline	18.9 (15.98-24.58)	16.9 (13.95-19)	16.65 (13.45-19.15)
	10 min after TRH/placebo	29.7 (20.55-36.75)	27.2 (14.1-36.9)	22.5 (16.6-38.53)
**Insulin (uIU/mL)**	Baseline	13.66 (11.14-19.83)	13.45 (10.23-17.51)	14.15 (9.273-17.58)
	60 min after OST	27.32 (14.84-43.89)	25.35 (15.74-41.69)	28.17 (17.7-43.33)
	90 min after OST	23.05 (13.28-33.65)	22.86 (14.78-40.77)	33.77 (20.03-54.25)
**Glucose (mg/dL)**	Baseline	89.5 (81.75-94.25)	87 (81.75-95.5)	84.5 (81-92.25)
	60 min after OST	112.5 (109-129.5)	119 (108.8-125.3)	122 (107.5-131.5)
	90 min after OST	117.5 (106.8-129)	118.5 (106.8-130)	118 (108.8-139)

Supplementary 4: Median (interquartile range) baseline ACTH, insulin and glucose concentrations, insulin and glucose concentrations 60 minutes after oral sugar administration, and insulin and glucose concentrations 90 minutes after oral sugar administration from each of the three OST.
